# Sunitinib facilitates metastatic breast cancer spreading by inducing endothelial cell senescence

**DOI:** 10.1186/s13058-020-01346-y

**Published:** 2020-09-29

**Authors:** Denian Wang, Fei Xiao, Zhongxue Feng, Min Li, Lingmiao Kong, Luping Huang, Yong’gang Wei, Hongyu Li, Fei Liu, Haili Zhang, Wei Zhang

**Affiliations:** 1 Department of Critical Care Medicine, State Key Laboratory of Biotherapy and Cancer Center, West China Hospital, Sichuan University and Collaborative Innovation Center of Biotherapy, No. 1, Ke Yuan 4th Road, Gao Peng Street, Chengdu, 610041 Sichuan People’s Republic of China; 2 Department of Intensive Care Unit of Gynecology and Obstetrics, West China Second University Hospital, Sichuan University, Chengdu, People’s Republic of China; 3 Department of Liver Surgery, West China Hospital, Sichuan University, Chengdu, People’s Republic of China; 4 Liver Transplantation Center, Beijing Friendship Hospital, Capital Medical University, Beijing, People’s Republic of China

**Keywords:** Sunitinib, Metastatic breast cancer (MBC), Receptor tyrosine kinase (RTK), Cell senescence, Metastasis

## Abstract

**Background:**

Sunitinib, a receptor tyrosine kinase (RTK) inhibitor that targets multiple receptors such as vascular endothelial growth factor receptors (VEGFRs), was approved for cancer treatment in 2006. However, it was unsuccessful in treating certain cancers, particularly metastatic breast cancer (MBC), and the mechanism underlying this “sunitinib resistance” remains unclear. Herein, we investigated whether the sunitinib-associated inferior survival benefit in MBC was due to sunitinib-induced endothelial cell (EC) injury or EC senescence.

**Methods:**

4T1 murine breast cancer cells were used as the main breast tumor model for it produces a highly metastatic solid tumor that can spontaneously metastasize to the lung, which closely mimics highly metastatic human breast cancer. Senescence-associated β-galactosidase (SA-β-Gal, immunohistochemistry [IHC]-staining), P16, P53, and P57 (immunoblotting) were used as markers of cell senescence. A protein array containing 25 senescence-associated chemokines and the transwell chemotaxis assay were used to examine whether sunitinib increases inflammatory chemokine secretion which attracts tumor cells via chemokinesis. Flow cytometry and IHC were used to detect whether the sunitinib-induced senescent ECs recruit cancer-associated inflammatory myeloid cells. Finally, the spontaneous metastatic model was used to monitor whether sunitinib causes the formation of “pre-metastatic niche” which promotes MBC to metastasize to the lungs.

**Results:**

We demonstrated that sunitinib induced a senescence-like endothelial cell (EC) phenotype. Inflammatory chemokine secretion and VCAM1 expression were significantly increased in senescent ECs, resulting in tumor cell (TC) chemotaxis and TC/EC interactions. Meanwhile, EC senescence caused loosening of EC junctions, facilitating TC transmigration through the endothelial barrier. Sunitinib-induced senescent ECs also recruited cancer-associated myeloid cells to form a “pre-metastatic niche”-like microenvironment. Alterations at the molecular level and in the tissue environment ultimately led to an increase in distant metastasis.

**Conclusion:**

Although sunitinib was designed to target the EC directly, the increase in tumor metastasis may ironically be due to sunitinib “correctly” playing its role. Our findings suggest that we should carefully weigh the pros and cons before using sunitinib and other antiangiogenic drugs that directly target the ECs.

## Background

Cancer cells require nutrients and oxygen to support their robust growth. Angiogenesis, the formation of new blood vessels from pre-existing vasculature, addresses those needs. Therefore, antiangiogenic treatment was considered a promising therapeutic strategy [1, 2]. Vascular endothelial growth factor (VEGF) plays a central role in this process through the activation of its receptor tyrosine kinases (RTKs), including VEGFR1, VEGFR2, and VEGFR3, on endothelial cells (ECs) [3, 4]. Sunitinib is a multitarget RTK inhibitor of VEGF receptors that targets VEGFR1, VEGFR2, and VEGFR3 as well as platelet-derived growth factor receptor (PDGFR), stem cell growth factor receptor, and FMS-like tyrosine kinase 3 [5–7]. Sunitinib has been approved for patients with advanced renal cell carcinoma, pancreatic neuroendocrine tumors, and gastrointestinal stromal tumors [8]. However, it was not as successful in certain cancer types, particularly metastatic breast cancer (MBC). Patients with MBC exhibited inferior progression-free survival (PFS) and overall survival (OS) when treated with chemotherapy plus sunitinib compared with patients treated with monochemotherapy [9–11]. In a phase II randomized trial, sunitinib treatment as a consolidation therapy after an objective response to taxane resulted in a shorter median PFS versus no treatment at all [12], and studies based on mouse models found that sunitinib could even accelerate the metastasis of breast cancer [6, 13]. A meta-analysis comprising 6 randomized controlled trials showed that sunitinib, either alone or in combination with chemotherapy, has no clinical benefit for patients with MBC [14]. Studies have attempted to investigate this “sunitinib resistance,” but the molecular basis underlying the ineffectiveness of sunitinib treatment in MBC is still lacking.

ECs not only play prominent roles in vasculature formation but also are critical components of the tumor microenvironment and the metastatic niche [15]. Because sunitinib targets almost all VEGF receptors and PDGFR expressed on the EC surface, we speculated that sunitinib might either attack the ECs directly or affect their normal functions both in the tumor vasculature and in the normal vasculature. Many anticancer drugs affect not only tumor cells (TCs) but also normal cells. In addition to their role in direct causing cell damage, anticancer drugs also induce cell senescence in the tumor microenvironment, including cancer cells [16] and noncancerous cells, such as fibroblasts, infiltrating immune cells, and ECs [17, 18]. Although drug-induced senescence could stimulate immunosurveillance, it could also promote the growth and aggressiveness of cancer cells by inducing the production of inflammatory cytokines, chemokines, and growth factors [19]. EC senescence was recently reported to facilitate metastasis [20]. Based on these studies, we hypothesized that sunitinib resistance, or a sunitinib-associated inferior survival benefit in MBC, may be due to sunitinib-induced EC injury or senescence.

Herein, we use 4T1 murine breast cancer cells as the main tool to study the effect of sunitinib on an MBC tumor model. We chose the 4T1 cell line because it produces a highly metastatic solid tumor that can spontaneously metastasize to the lung, which closely mimics highly metastatic human breast cancer [21, 22]. Our data suggest that sunitinib does not cause direct EC damage but induces a senescence-like EC phenotype. The presence of senescent ECs increased inflammatory chemokine secretion and VCAM1 expression, which attracted tumor cells (TCs) of MBC to ECs and facilitated the TC/EC interaction. Meanwhile, the expression of the key junction molecule VE-cadherin was reduced in the senescent ECs, resulting in the opening of EC junctions, which favors TC transmigration to the endothelial barrier. In addition, senescent ECs recruited cancer-associated inflammatory myeloid cells, including neutrophils and macrophages, which contributed to the formation of a “premetastatic niche”-like microenvironment. These molecular and environmental changes ultimately led to an increase in lung metastasis. Our study provides a possible explanation for the phenomenon that sunitinib has no clinical benefit in patients with MBC and improves our understanding of the mechanism underlying the observed drug resistance to antiangiogenic therapies.

## Materials and methods

### Cells and culture conditions

Mouse mammary cancer 4T1 and human mammary cancer MDA-MB-231 cell lines were from ATCC and routinely cultured in complete Dulbecco’s Eagle’s medium (DMEM). Human umbilical vein endothelial cells (HUVECs) were isolated from the umbilical cords and routinely grown in EGM-2 (Lonza, Cat. CC-3162). Human skin fibroblasts (HSFs) for co-culturing with HUVECs were isolated from surgical specimens and routinely grown in DMEM supplemented with 10% fetal bovine serum at 37 °C and 5% CO_2_.

### Antibodies and reagents

Antibodies for P57 (Cat. Ab75974), P16 (Cat. Ab189034), VCAM-1 (Cat. Ab134047), and CD11b (Cat. Ab133357) were from Abcam; antibody for P53 (Cat. #2524) and Senescence β-Galactosidase Staining Kit (Cat. #9860) were from Cell Signaling Technology. Antibody for β-actin (Cat. sc-47,778) was from Santa Cruz. Alexa Fluor® secondary antibodies were from Life Technology. Sunitinib (Cat. S7718) was from Selleck. TRIzol Reagent was obtained from Invitrogen. Mouse CCL6 ELISA Kit (ab193719), mouse complement C5a ELISA Kit (ab193718), mouse Chemerin ELISA Kit (ab204520), and mouse IL-16 ELISA Kit (ab201282) were from Abcam.

### Cell viability assay

The CCK-8 cell viability assay was performed according to the manufacturer’s instructions. In brief, 6 replicates of 5000 cells/well (HUVECs) had been plated in 96-well plates and allowed to attach, then grown in EGM-2 at 37 °C overnight. Pre-incubate the plate for 24 h in a humidified incubator (37 °C, 5% CO_2_). Add 10 μl of various concentrations of sunitinib into the culture media in the plate. Incubate the plate for 12 h, and CCK-8 solution to each well of the plate. Incubate the plate for 1–4 h, and measure the absorbance at 450 nm using a microplate reader.

### Chemokine array

The Human Chemokine Array (Cat. ARY017) was performed according to the manufacturer’s instructions. HUVECs were cultured and treated with sunitinib (20 μM) for 24 h, and the cell culture supernatant was collected. Pipette 2.0 ml of Array Buffer 6 (blocking buffer) into each well of the 4-Well Multi-dish to be used. Using flat-tip tweezers, remove each membrane to be used from between the protective sheets and place in a well of the 4-Well Multi-dish. The number on the membrane should be facing upward. Incubate for 1 h on a rocking platform shaker. Orient the tray so that each membrane rocks end to end in its well. While the membranes are blocking, prepare samples by adding up to 1 ml of each sample to 0.5 ml of Array Buffer 4 in separate tubes. Add 15 μl of reconstituted Detection Antibody Cocktail to each prepared sample. Mix and incubate at room temperature for 1 h. Aspirate Array Buffer 6 from the wells of the 4-Well Multi-dish and add the prepared sample/antibody mixtures. Incubate overnight at 2–8 °C on a rocking platform shaker. After the incubation is completed, carefully wash the membrane and perform the Streptavidin-HRP staining. The positive signals seen on developed film can be quickly identified by placing the transparency overlay on the array image and aligning it with the pairs of reference spots in three corners of each array.

### Real-time RT-PCR

Total RNA was isolated using TRIzol Reagent. Reverse transcription was performed with a Superscript II Two-Step RT-PCR Kit (Invitrogen). PCR was performed using SYBR® Green PCR Master Mix (Applied Biosystems). Gene expression was normalized to GAPDH.

### Western blot analysis

Cell extracts were separated by SDS-PAGE, electro-transferred onto polyvinylidene fluoride membranes, and blocked in 5% nonfat milk in Tris-buffered saline/0.01% Tween 20 for 2 h. Blots were incubated at 4 °C in Tris-buffered saline with primary antibody (dilution according to the manufacturer’s instruction), followed by 1 h incubation with horseradish peroxidase-conjugated secondary antibody and detected by a chemiluminescence kit (Millipore, Cat. WBKLS0100).

### Tumor study

All animal experiments were approved by the Animal Ethics Committee of Sichuan University and performed according to the institutional and national guidelines. Balb/c or NOD-SCID mice (6–8 weeks, 20–25 g, housed in specific pathogen-free [SPF] conditions) were used in this study. 4T1 or MDA-MB-231 mammary cancer were established in Balb/c or SCID mice, respectively. Mice were injected orthotopically with 2.5 × 10^6^ cells under the mammary fat pad. The tumor size was measured every 3 days after the inoculation of tumor cells. For establishing the PDX model, the clinical MBC samples from patients with invasive ductal carcinoma were collected. SCID/NOD mice were pretreated with sunitinib or vehicle for 2 weeks, and the MBC tumor tissues were orthotopically implanted under the mammary pads. The tumor volume was calculated by the following formula: volume (mm^3^) = 1/2 × length (mm) × width (mm) × width (mm). Mice were anesthetized and euthanized at the end point of the tumor experiment (when the largest tumor reached about 1500 mm^3^ according to the ethical standards for animal welfare) and perfused transcardially with 4% PFA in PBS for 10 min. The number of surface metastatic lesions in the lungs was counted manually and presented as scatter plots with mean ± SEM.

### Immunohistochemical analysis of protein expression

The tumor and tissue samples were removed and post-fixed with 4% paraformaldehyde for 24 h, embedded in paraffin, and sectioned at a 5-μm thickness. Sections were stained with primary antibodies, and signals were developed by incubating the sections with DAB chromogen (brown) and counterstaining with hematoxylin (blue). Protein expression was scored as follows: 0 points, no positive cells; 1 point, < 10% positive cells; 2 points, 10–50% positive cells; 3 points, 51–80% positive cells; and 4 points, > 80% positive cells. The staining intensity was rated as follows: 1 point, weak staining; 2 points, moderate intensity; and 3 points, strong intensity. Points were added to generate overall scores. The staining was scored by two blinded observers.

### Miles assay (the in vivo permeability assay)

After 2 weeks of saline or sunitinib treatment, Evans Blue (EB) was injected into the tail veins of mice (6–8 weeks of age). After 30 min, VEGF (50 ng in 10 μl of saline) was injected into the dermis to induce permeability. After another 30 min, mice were then sacrificed and an 8-mm biopsy located around the injection site (using the site of injection as the center point for the biopsy) was removed. Permeability was quantitated by monitoring the elution of the EB dye in formamide at 56 °C overnight, and the absorbance was measured at 630 nm. The data were acquired from 2 skin samples from each mouse, and 5 mice per group were analyzed.

### Bronchus bronchoalveolar lavage assay

After 2 weeks of sunitinib treatment, bovine serum albumin (BSA) was injected intravenously. Mice were then sacrificed, and a bronchoalveolar lavage (BAL) of the right lung was performed twice with 400 μl of saline each. Two hundred fifty microliters was pooled from each BAL aliquot, and BAL and plasma BSA concentrations were determined by ELISA. Permeability was assessed by calculating the BSA/BAL and BSA/plasma ratios. The amount of protein leakage in the lung was consistent with our previous data obtained from the EB leakage assay.

### Statistics

The statistical power was calculated to determine the *n*-number of each group. No randomization was applied because all mice used were genetically defined, inbred mice. When comparing the two groups for which a Gaussian distribution was not assumed, the unpaired, 2-tailed nonparametric Mann-Whitney *U* test was used; when a Gaussian distribution was assumed, the unpaired, 2-tailed parametric *t* test with Welch’s correction was used. A *p* value < 0.05 was considered statistically significant: *p < 0.05, **p < 0.01, and *** *p* < 0.001. Survival outcomes were analyzed using Kaplan-Meier survival curves, and the significance of the differences was assessed using the log-rank test. The log-rank test was used to compare the two groups. *p* values of 0.05 or less were considered to denote a significant difference.

## Results

### Sunitinib induced a senescence-like phenotype in cultured ECs

**Fig. 1:**
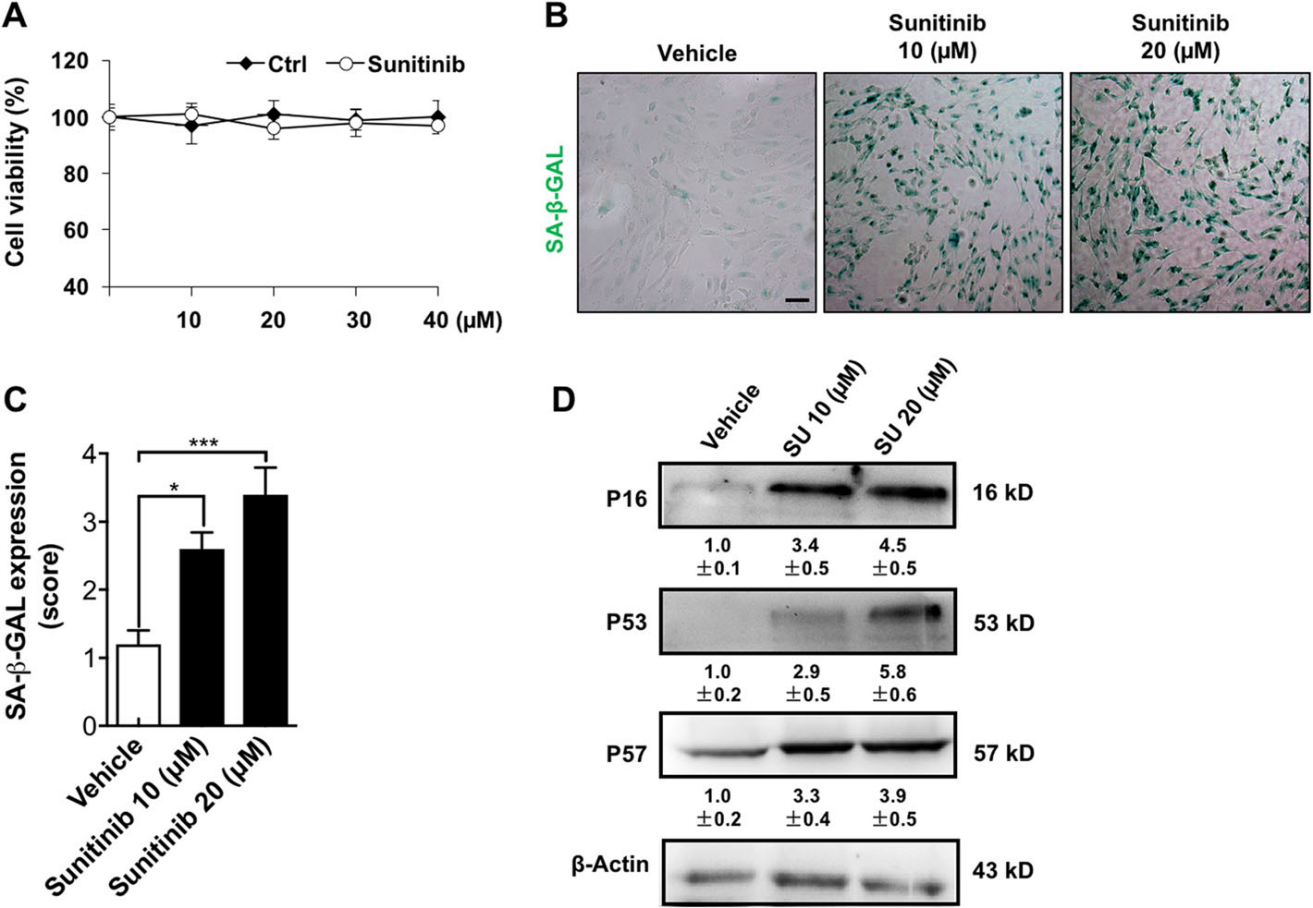
Sunitinib induced a senescence-like phenotype in cultured ECs. **a** HUVEC viability was measured after the treatment of sunitinib. **b** HUVECs were stained for SA-β-GAL (green) after the treatment of sunitinib. **c** The score of SA-β-GAL expression was quantified using 10 randomly chosen fields from two experiments as described in the “Materials and methods” section. The data were presented as columns with mean ± SEM. Significance was assessed by unpaired *t* test with Welch’s correction. **d** HUVECs were treated with sunitinib and then subjected to WB assay. The densities of the bands for P16, P53, and P57 (referred to β-actin) were presented relative to that of the control (means ± SD, *n* = 3; the mean level in the control group was set to 1.0)

As mentioned above, nearly all the targets of sunitinib are on the surface of ECs. Thus, sunitinib may damage the blood vessel system, which may favor the spread of cancer cells. First, we tested whether sunitinib caused direct damage to normal ECs. The cytotoxicity assay showed that sunitinib exhibited no direct killing activity on cultured human umbilical vascular endothelial cells (HUVECs) (Fig. 1a). We then asked whether sunitinib affected the status of EC shifting to a senescence-like phenotype. Senescent cells express distinctive molecular markers, including senescence-associated β-galactosidase (SA-β-Gal), P16, P53, and P57 [23, 24]. Sunitinib treatment markedly increased the expression of SA-β-Gal in ECs (Fig. 1b, c) and induced the expression of P57, P53, and P16 in a dose-dependent manner (Fig. 1d). The results suggest that sunitinib may induce the shift of ECs into a senescence-like phenotype.

### Sunitinib induced a senescence-like EC phenotype in vivo

**Fig. 2:**
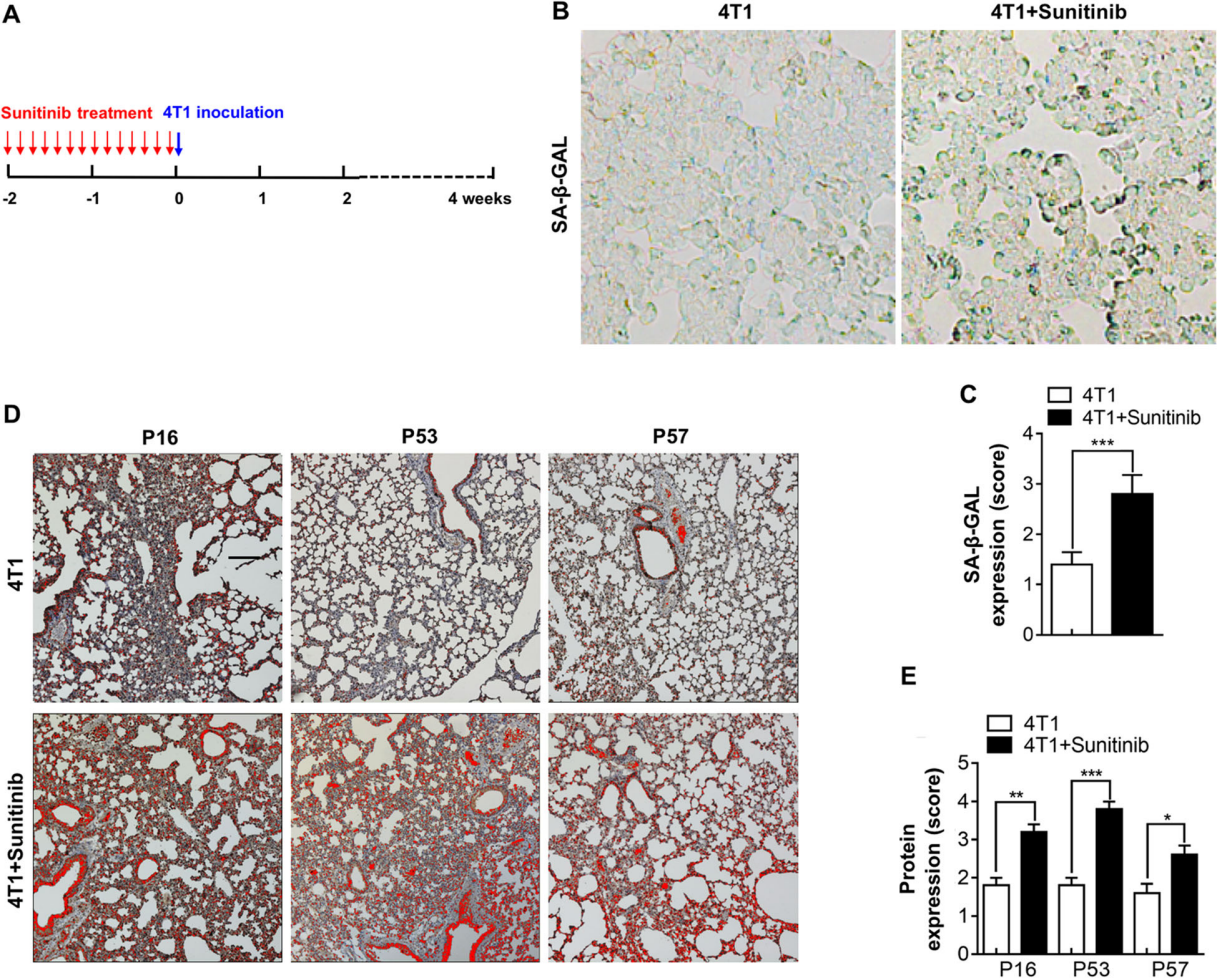
Sunitinib induced a senescence-like EC phenotype in vivo. **a** Balb/c female (6–8 weeks, 20–25 g, housed in SPF conditions, *n* = 5) mice were treated with sunitinib (100 mg/kg/day, intragastric administration) for 2 weeks (14 doses total) and inoculated with 1 × 10^6^ 4T1 cells. **b** The representative images showed the positive staining of SA-β-GAL (marked by pseudo-color using the software ImagePro Plus) in the lungs from the 4T1 tumor-bearing mice. **c** The scores of SA-β-GAL expression were quantified using 25 randomly chosen fields from 5 mice of each group as described in the “Materials and methods” section. **d** The lungs from 4T1 tumor-bearing mice were stained for P16, P53, and P57 (marked by pseudo-color using the software ImagePro Plus). **e** The expression scores of P16, P53, and P57 were quantified using 25 randomly chosen fields from 5 mice of each group. Data were presented as columns with mean ± SEM. Significance was assessed by unpaired *t* test with Welch’s correction

To determine whether sunitinib induced a senescence-like EC phenotype in vivo, we established an orthotopic breast cancer model by injecting 4T1 cells under the mammary fat pad of BALB/c mice. Considering the lung contains an abundant number of microvessels and ECs and is one of the major distal metastatic sites of breast cancer, we analyzed lung samples from tumor-bearing mice treated with or without sunitinib. Before TC inoculation, mice were administered sunitinib or vehicle for 2 weeks, as shown in Fig. 2a. The expression of SA-β-GAL were significantly increased in the lungs from sunitinib-treated mice (Fig. 2b, c). In addition, the expression levels of the senescence markers P16, P53, and P57 were also increased in lungs from the sunitinib-administered mice but not in mice treated with the vehicle (Fig. 2d. e). According to the results, sunitinib induced a senescence-like EC phenotype in vivo.

### Sunitinib-induced senescent ECs exhibited increased inflammatory chemokine secretion and attracted tumor cells via chemokinesis

**Fig. 3:**
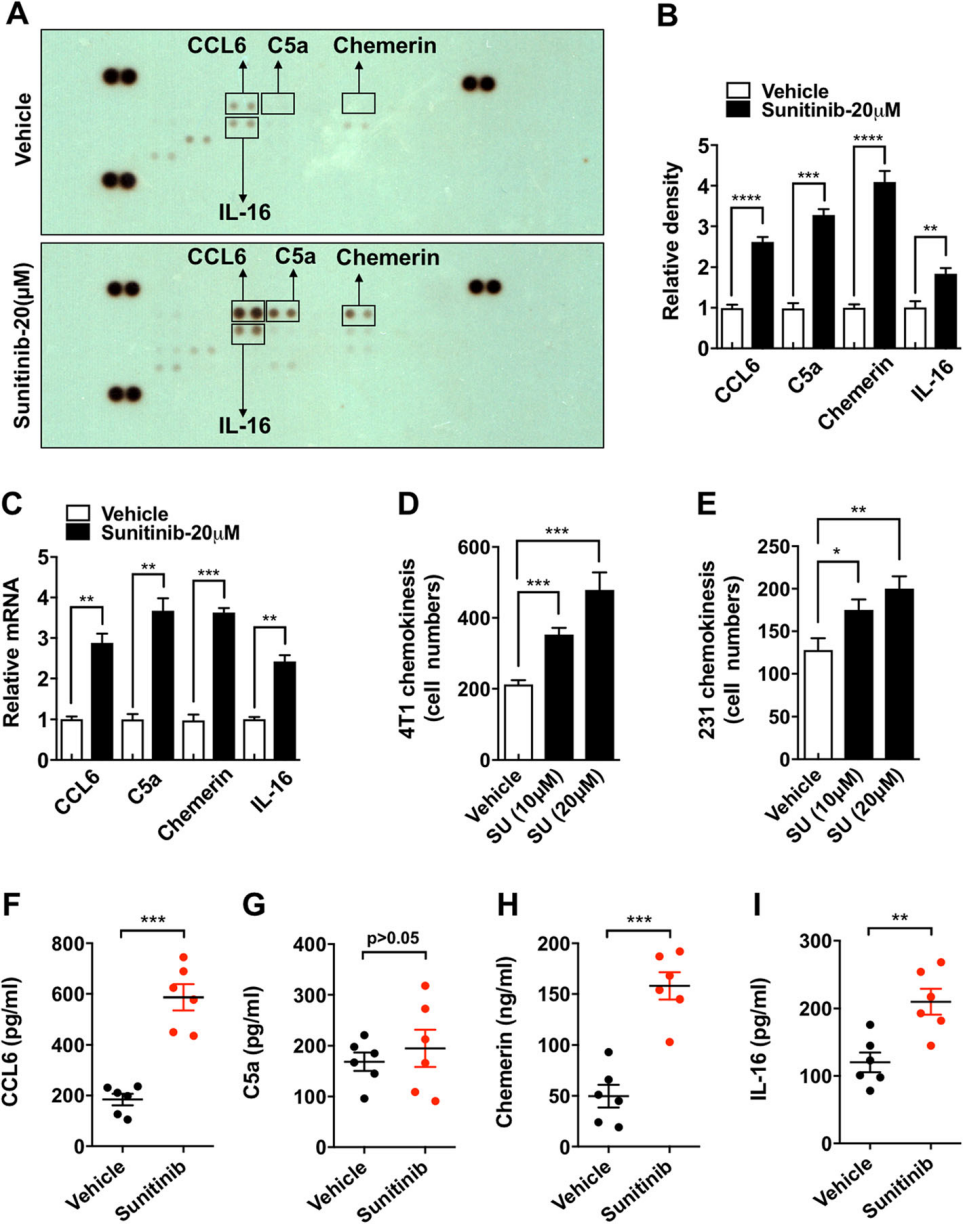
Sunitinib-induced senescent ECs exhibited increased inflammatory chemokine secretion and attracted tumor cells (TCs) via chemokinesis. **a** HUVECs were treated with vehicle (0.1% DMSO) or sunitinib (20 μM) for 24 h, and the supernatant was collected and subjected to the cytokine array containing 25 inflammatory chemokines. **b** The staining densities of CCL6, C5a, chemerin, and IL-16 were presented relative to that of the control. The mean level in the control group was set to 1.0; *n* = 4. **c** HUVECs were treated with sunitinib (20 μM) or vehicle (0.1% DMSO) for 24 h, and the relative mRNA levels of CCL6, C5a, chemerin, and IL-16 were measured by real-time RT-PCR (*n* = 5). **d**, **e** 4T1 and MDA-MB-231 cells were cultured in the upper chamber, and HUVECs pre-treated with sunitinib (or vehicle) were cultured in the lower chamber. The number of cells that crossed the *Transwell* membrane toward the lower chamber was counted (*n* = 5). Data were presented as columns with mean ± SEM. **f**–**i** Blood samples from mice administrated with sunitinib (100 mg/kg) were harvested. The concentration of CCL6 (**f**), C5a (**g**), chemerin (**h**), and IL-16 (**i**) was determined by ELISA. Significance was assessed by unpaired *t* test with Welch’s correction

Senescent cells could change the cellular microenvironment by secreting inflammatory chemokines [25, 26]. Using a protein array containing 25 chemokines related to the senescence-associated secretory phenotype (SASP), we found that four chemokines, CCL6, complement component C5a, chemerin, and IL16, were increased upon sunitinib stimulation (Fig. 3a, b). The upregulation of these 4 genes was confirmed by real-time RT-PCR (Fig. 3c). Because TCs often express chemokine receptors [27], we assumed that TCs might be chemotactically attracted to sunitinib-stimulated ECs. The transwell chemotaxis assay showed that 4T1 and MDA-MB-231 cells, the canonical mouse and human MBC cell line, respectively, had higher migration rates when HUVECs treated with sunitinib were cultured in the bottom chamber (Fig. 3d, e). To determine whether the 4 cytokines were also upregulated in vivo, the plasma level of them were detected using ELISA assay. We found that the administration of sunitinib significantly increased the plasma levels of CCL6, chemerin, and IL-16, but not that of C5a, suggesting that CCL6, chemerin, and IL-16 may be responsible for the sunitinib-induced chemotaxis.

### Sunitinib-induced senescent ECs exhibited increased VCAM1 expression and a greater TC/EC interaction

**Fig. 4:**
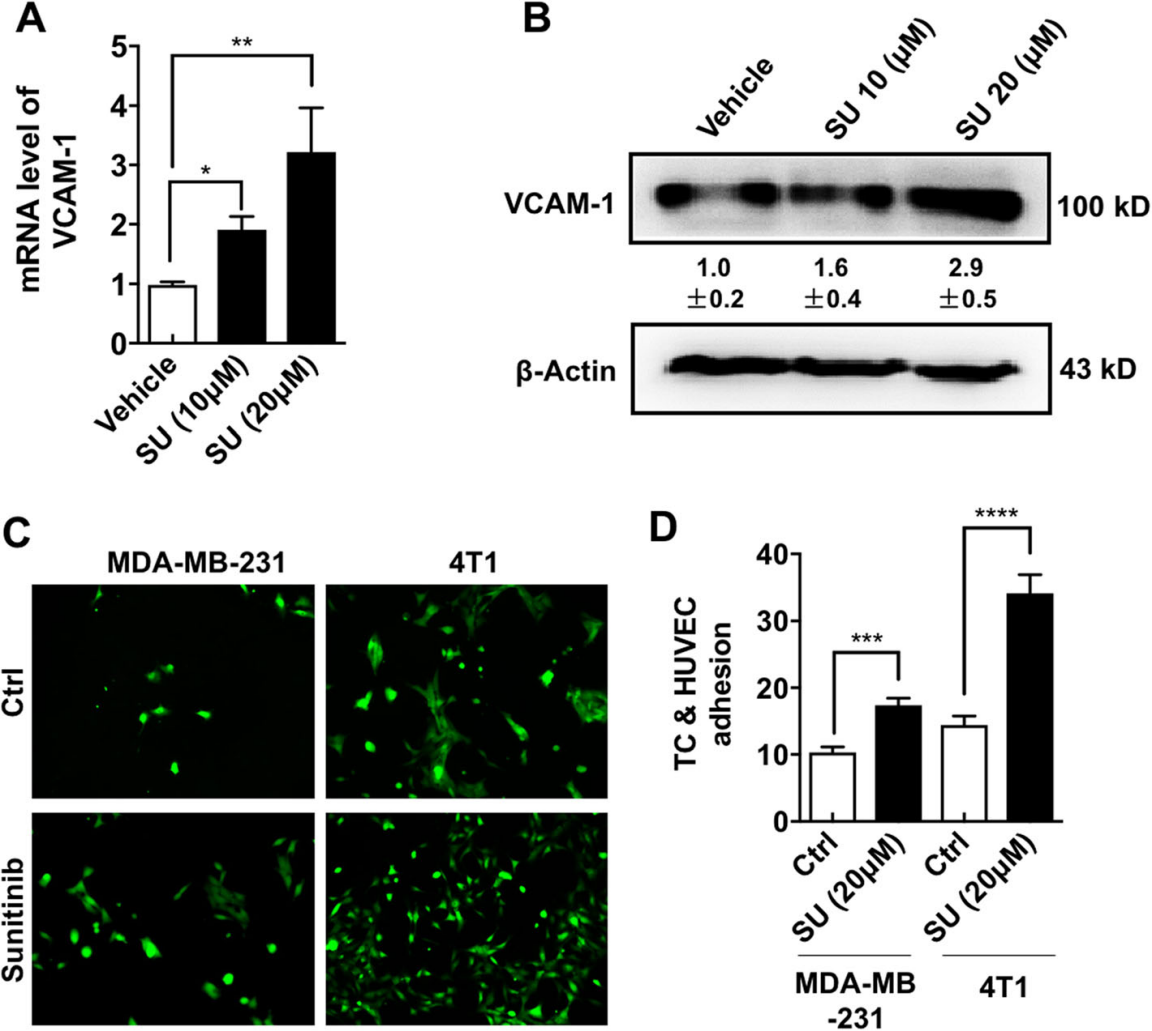
Sunitinib-induced senescent ECs exhibited increased VCAM1 expression and a greater TC/EC interaction. **a** The mRNA level of VCAM-1 in HUVECs treated with vehicle or sunitinib was measured by real-time RT-PCR. **b** HUVECs treated with vehicle or sunitinib were subjected to WB assay to test the VCAM-1 expression (the mean level in the control group relative to β-actin was set to 1.0; *n* = 3). **c** EGFP-transfected 4T1 or MDA-MB-231 cells were co-cultured with HUVEC monolayer that was pre-treated with vehicle or sunitinib (20 μM). The cells that did not adhere to the ECs were washed using PBS. **d** The number of 4T1 or MDA-MB-231 cells that adhered to HUVECs was quantified using 10 randomly chosen fields from two experiments. Data were presented as columns with mean ± SEM. Significance was assessed by unpaired *t* test with Welch’s correction

Senescence-related inflammatory chemokines could increase the expression of leukocyte adhesion molecules such as vascular cell adhesion molecule-1 (VCAM1) on the surface of ECs [28]. RT-PCR and Western blot (WB) assays showed that the mRNA and protein levels of VCAM1 were significantly increased in sunitinib-treated HUVECs, respectively (Fig. 4a, b). The increased VCAM1 expression in ECs may promote the interaction of TCs and ECs, which facilitates tumor metastasis [29, 30]. Indeed, sunitinib markedly induced 4T1 and MDA-MB-231 cells to adhere to the EC monolayer (Fig. 4c, d). Based on these results, the sunitinib-induced senescence-like EC phenotype may attract TCs and promote the subsequent TC/EC interaction, which facilitates cancer metastasis.

### Sunitinib-induced EC senescence resulted in vessel leakage and TC extravasation

**Fig. 5:**
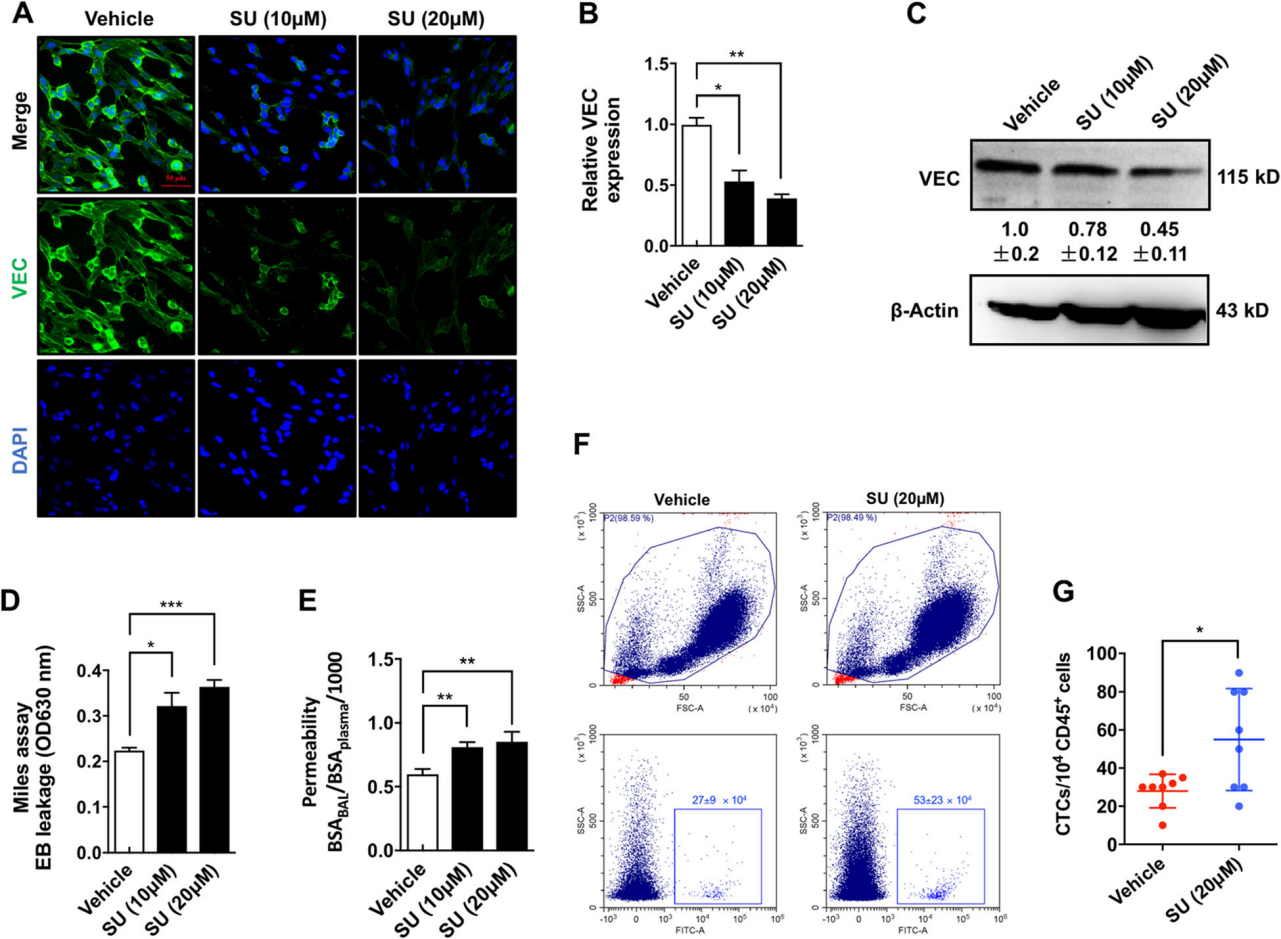
Sunitinib-induced EC senescence resulted in vessel leakage and TC extravasation. **a** The HUVECs treated with vehicle or sunitinib were immunofluorescent stained for VEC (green). **b** VEC expression was quantified using 10 randomly chosen fields from two experiments using ImagePro Plus (the mean level in the vehicle group was set to 1.0; *n* = 10). **c** HUVECs treated with vehicle or sunitinib were subjected to WB assay (means ± SD; *n* = 3). **d** The Miles assay was performed as described in the “Materials and methods” section. The Evans Blue (EB) leakage (OD 630 nm) was quantified (*n* = 10 mice). **e** Bronchus bronchoalveolar lavage assay to examine the permeability of the lungs from mice treated with vehicle or sunitinib. **f**, **g** Balb/c female mice were treated with sunitinib (100 mg/kg/day) for 2 weeks and inoculated with 1 × 10^6^ EGFP-transfected 4T1 cells. The number of green fluorescence-positive CTCs in the blood of tumor-bearing mice was counted using flow cytometry, and data were presented as scatter plots with mean ± SEM (*n* = 8). Significance was assessed by the Mann-Whitney test

The senescent phenotype affects the function of EC junctions by downregulating vascular endothelial cadherin (VEC), a major component of adherens junctions at cell-cell contacts of ECs [31]. Fluorescent staining showed that sunitinib reduced the expression of VEC in ECs (Fig. 5a, b), and this effect was further supported by WB assay to be dose-dependent (Fig. 5c). The sunitinib-induced downregulation of VEC resulted in hyperpermeability, which was evidenced by both in Miles assay (Fig. 5d) and in bronchus bronchoalveolar lavage assay (Fig. 5e). Flow cytometry analysis (Fig. 5f, g) showed that the number of circulating tumor cells (CTCs) in the blood of sunitinib-treated mice was increased compared to that in vehicle-treated mice, suggesting that sunitinib-induced endothelial leakage increased TC extravasation and might lead to distant metastasis.

### Sunitinib-induced senescent ECs recruited cancer-associated inflammatory myeloid cells

**Fig. 6:**
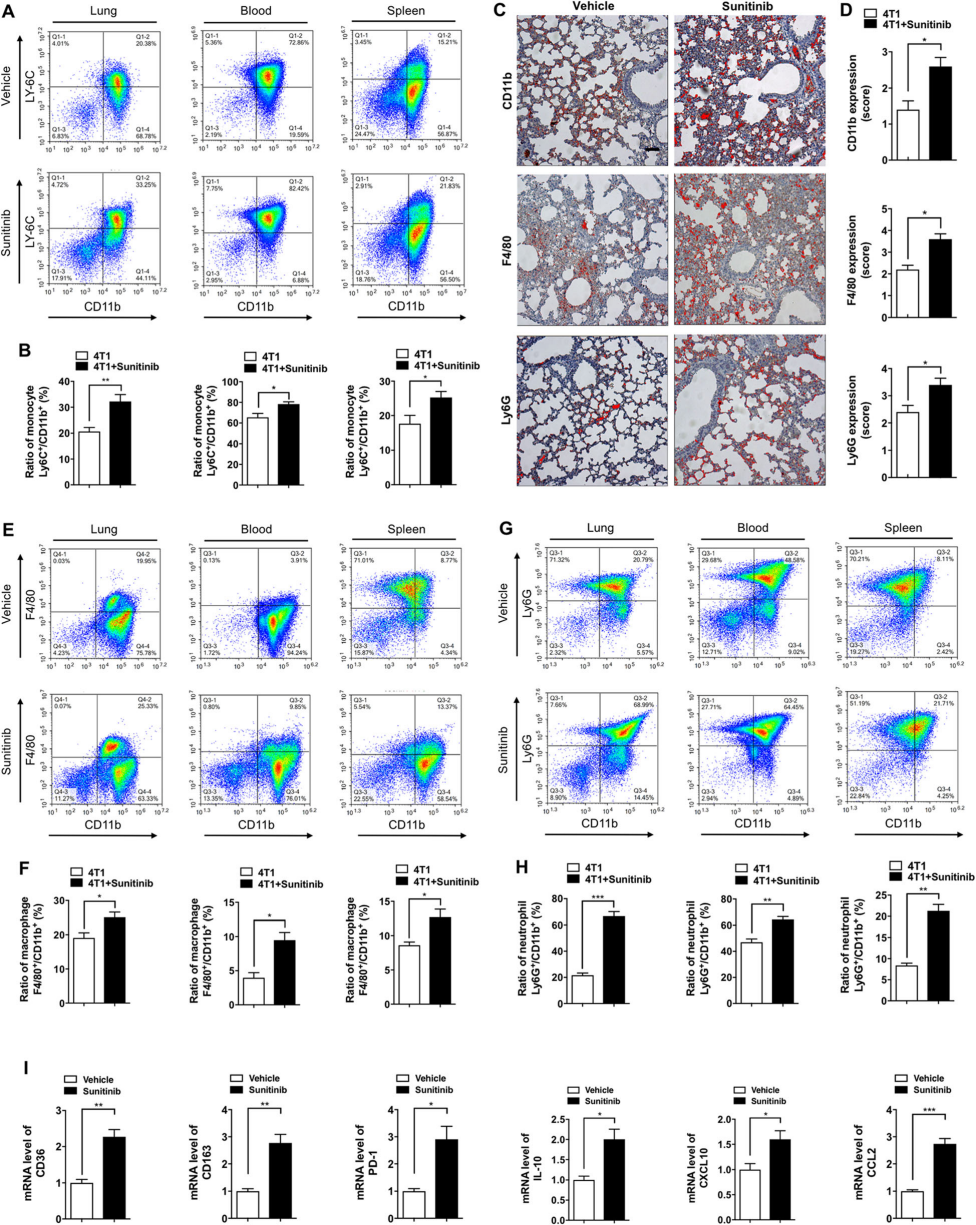
Sunitinib-induced senescent ECs recruited cancer-associated inflammatory myeloid cells. **a**, **b** Balb/c female mice were pre-treated with sunitinib (100 mg/kg/day) for 2 weeks, followed by inoculation with 1 × 10^6^ transfected 4T1 cells. The monocyte population (gating strategy: Ly6C^+^/CD11b^+^) in the lung, blood, and spleen were quantified by flow cytometry. Data were presented as columns with mean ± SEM (*n* = 3). **c** The lung samples were immunostained for CD11b, F4/80, and Ly6G (marked by pseudo-color using ImagePro Plus). **d** The expression score of CD11b, F4/80, and Ly6G was quantified as described in the “Materials and methods” section. **e**–**h** The macrophage (F4/80^+^/CD11b^+^) and neutrophil (Ly6G^+^/CD11b^+^) population in the lung, blood, and spleen were quantified by flow cytometry, and data were presented as columns with mean ± SEM (*n* = 3). **i** The infiltrating macrophages were sorted using flow cytometry and subjected to semi-quantitative RT-PCR to detect the level of CD36, CD163, PD-1, IL10, CXCL10, and CCL2. Significance was assessed by unpaired *t* test with Welch’s correction

EC senescence was reported to promote cancer-associated inflammatory cell infiltration [20]. We have shown that the sunitinib-induced senescent EC phenotype increased inflammatory chemokine secretion (Fig. 3a–c). Thus, these chemokines may recruit tumor-associated myeloid cells and trigger the formation of a premetastatic niche. To test this, mice were treated with sunitinib and orthotopically inoculated mice with 4T1 cells as indicated in Fig. 2a. Because MBCs have a higher frequency of lung metastasis, we analyzed the lung tissues and detected significant infiltration of the CD11b^+^ population in the lungs after 2 weeks of sunitinib treatment. The flow cytometry results showed that the increased population was CD11b^+^/Ly6C^+^ (Fig. 6a, b), indicating that the infiltrating cells are monocytes. The increased monocyte count in the lungs was associated with an increase in monocytes in the blood and spleen of sunitinib-treated mice (Fig. 6a, b). We analyzed the subpopulation of the recruited monocytes using the macrophage marker F4/80 and the neutrophil marker Ly6G. Immunohistochemistry analysis demonstrated that the monocyte population recruited to the lungs contains both macrophages and neutrophils (Fig. 6c, d). Flow cytometry showed that the increased numbers of macrophages and neutrophils in the lung were associated with the blood and spleen (Fig. 6e–h).

### The “premetastatic niche”-like environment induced by sunitinib promoted MBC to metastasize to the lungs

**Fig. 7:**
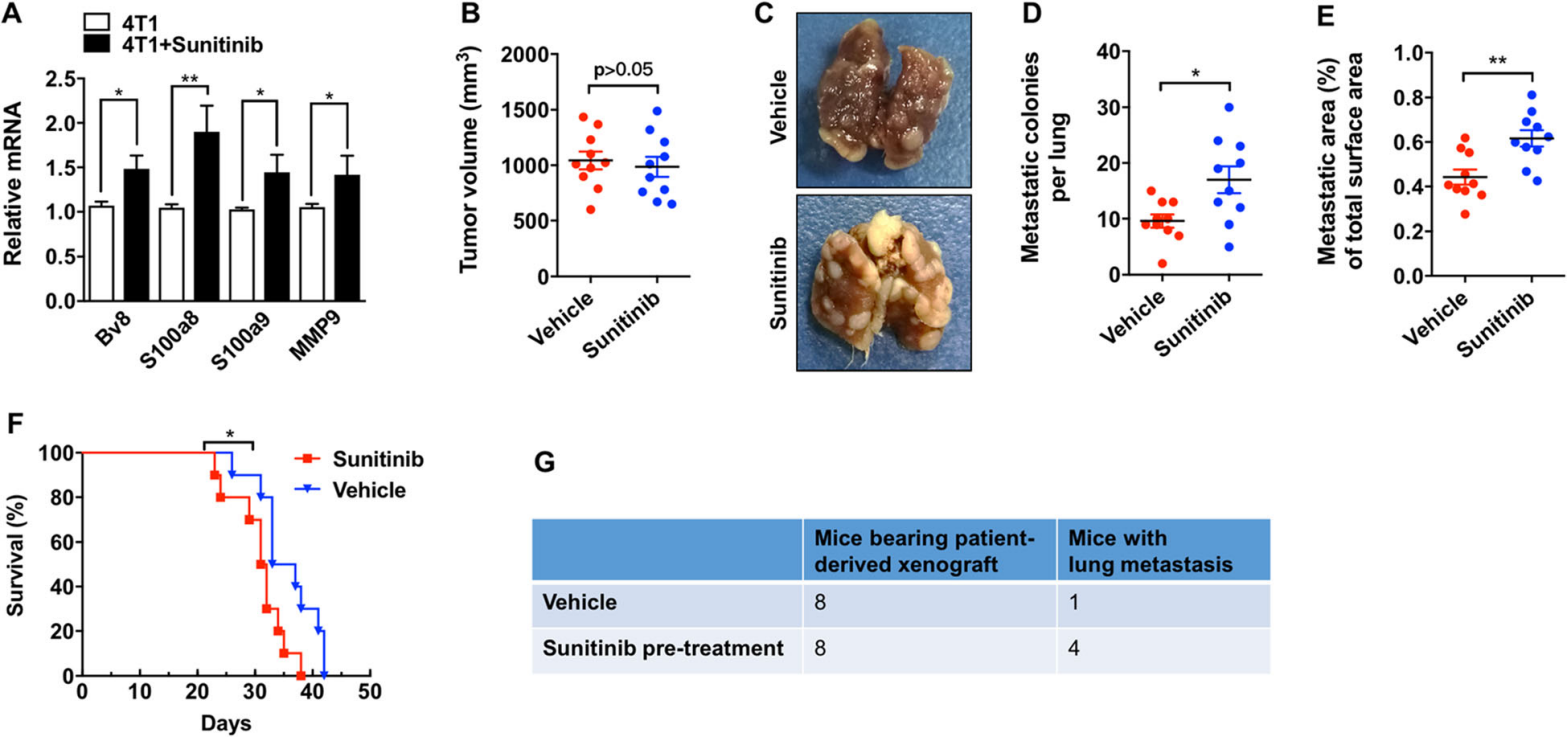
The premetastatic niche environment induced by sunitinib promoted MBC to metastasize to the lungs. **a** The relative mRNA levels of Bv8, S100a8, S100a9, and MMP9 were measured by real-time RT-PCR (*n* = 5). **b** Balb/c female mice were pre-treated with sunitinib (100 mg/kg/day) or saline for 2 weeks, followed by orthotopic inoculation with 1 × 10^6^ transfected 4T1 cells. The tumor volume was measured and presented as scatter plots with mean ± SEM (*n* = 10). **c** Representative images of the lungs with metastatic tumor colonies. **d**, **e** The number of surface metastatic colonies and the area of the metastatic lesion were quantified and presented as scatter plots with mean ± SEM (*n* = 10). **e** Kaplan-Meier survival analysis of the mice pretreated with sunitinib or saline. **f** Lung metastatic ability assay using patient-derived xenografts (*n* = 8). Significance was assessed by unpaired *t* test with Welch’s correction (**b**, **d**) and log-rank test (**e**)

The increased inflammatory chemokine secretion, the upregulation of VCAM1, the senescence-induced vascular leakiness, and the recruitment of cancer-associated myeloid cells all contribute to the formation of a “premetastatic niche”-like environment because these conditions provide a hospitable setting for the colonization of disseminated cancer cells [32, 33]. Thus, we then detected the expression of S100a8, S100a9, Bv8, and Mmp9, prometastatic proteins that promote colonization at metastasis sites [32, 34]. RT-PCR analysis showed that the mRNA levels of Bv8, S100a8, S100a9, and Mmp9 were significantly higher in the lungs of tumor-bearing mice after 2 weeks of sunitinib treatment than those in tumor-bearing control mice (Fig. 7a). The upregulation of these genes may favor the metastasis of cancer cells to the lungs.

To test this, sunitinib was administered to mice for 2 weeks before 4T1 cell inoculation. Sunitinib pretreatment did not affect primary tumor growth (Fig. 7b); however, it significantly increased lung metastasis compared to that in untreated mice, as evidenced by the increased number and surface area of metastatic colonies on the surface of the lungs in sunitinib-treated mice (Fig. 7c–e). The Kaplan-Meier survival analysis showed that tumor-bearing mice with sunitinib pretreatment had a worse survival rate than untreated mice (Fig. 7f). To further test this hypothesis, we established a patient-derived xenograft (PDX) model using clinical MBC samples from patients with invasive ductal carcinoma. SCID/NOD mice were pretreated with sunitinib or vehicle for 2 weeks, and the MBC tumor tissues were orthotopically implanted under the mammary pads. Four weeks after tumor inoculation, we examined the mice and found that compared to vehicle treatment, pretreatment with sunitinib markedly increased the chance of lung metastasis (Fig. 7g). In addition, we evaluated the phenotype of the macrophages that infiltrated into the lungs. The tumor-associated macrophages are the major immune cells that can promote the establishment of metastases. The infiltrating macrophages from the vehicle- or sunitinib-treated mice were isolated using flow cytometry sorting (F4/80^+^ cells). The semi-quantitative RT-PCR showed that the sunitinib treatment significantly increased the expression levels of CD36, CD163, PD-1, IL10, CXCL10, and CCL2. The upregulation of the immune suppression markers indicates the infiltrating macrophages were polarized into M2, which may facilitate the tumor metastasis.

## Discussion

Sunitinib is an antiangiogenic tyrosine kinase inhibitor of multiple targets [5–7]. Although it has been used to treat cancer patients for years, it is not as successful in certain types of cancers, particularly MBC [9–12]. It could even accelerate breast cancer metastasis in certain settings [6, 13]. Researchers have tried to determine why sunitinib treatment is unsuccessful in MBC, but the underlying molecular mechanism is still unclear. Herein, we show that sunitinib induces a senescence-like EC phenotype in vitro and in vivo. Inflammatory chemokine secretion and VCAM1 expression are significantly increased in senescent ECs, resulting in TC chemotactically attracted to the senescent EC and TC/EC interaction. Meanwhile, VEC expression is markedly decreased in senescent ECs, which causes EC junction loosening and promotes TC transmigration through the endothelial barrier. Sunitinib also induces the senescent EC phenotype in distant organs as well as at primary tumor sites. Senescent ECs recruit cancer-associated inflammatory myeloid cells, which facilitate the formation of a “premetastatic niche”-like environment in the distant organ. Ultimately, alterations at the molecular level and in the tissue environment lead to an increase in distant metastasis.

Because sunitinib is a multitarget antiangiogenic drug, it is theoretically considered a broad-spectrum antitumor drug. Sunitinib was approved for treating gastrointestinal stromal tumors and advanced renal cell carcinoma as early as 2006 [35]. Since then, people have been working to broaden the scope of its use. Unfortunately, more than a decade has passed, and there have been more than 2000 studies on the use of sunitinib to treat a variety of cancers, but its scope of clinical application has only increased by one (advanced pancreatic neuroendocrine tumors). The clinical trials showed that sunitinib, either alone or in combination with chemotherapy, has no significant clinical benefit in patients with other types of cancers, particularly in patients with MBC [9–12, 14]. Some studies reported that sunitinib even accelerated the metastasis of breast cancer [13, 36–38]. The continuous failure of sunitinib in MBC suggests that there is a great gap between the role we thought sunitinib might play and the role it actually has. Clarifying the exact effects of sunitinib and elucidating the underlying mechanisms are crucial for us to better understand how this antiangiogenic therapy affects tumor-associated microenvironments and TC behavior and to decide under what circumstances this treatment is suitable for cancer.

Several studies have started to explore why MBC is resistant to sunitinib treatment [39–41]. Zhang et al. [39] and Chinchar et al. [40] found that sunitinib increased the population of breast cancer stem cells. According to Braga et al.’s report [41], the primary resistance of MBC to sunitinib was likely mediated by the upregulation of hypoxia-responsive genes. Chung and colleagues reported that sunitinib induced loss of VE-cadherin and increased tumor cell intravasation [42]. These studies mainly focused on the effect of sunitinib on TCs themselves. In the present study, we analyzed this “sunitinib resistance” from another angle. Our data suggest that sunitinib effects tumor metastasis by affecting ECs, one of the most important components of the tumor microenvironment. ECs are not only the main building blocks of the tumor vasculature but also responsible for remodeling the tumor microenvironment through the paracrine release of endothelial-derived factors and the recruitment of inflammatory myeloid cells [15]. Changes in ECs themselves, such as a shift toward a senescence-like EC phenotype, can affect the integrity of the blood vessels, which may facilitate the passage of TCs through the endothelial barrier. The abnormally expressed molecules by senescent ECs attract TCs via chemokinesis and recruit cancer-associated inflammatory cells to form a “premetastatic niche”-like microenvironment in distant organs, which ultimately leads to cancer metastasis. The EC itself is the designed target for antiangiogenic drugs, especially for sunitinib (which targets VEGFR1, VEGFR2, VEGR3, and PDGFR, all of which are expressed on the surface of ECs). The sunitinib-affected ECs, however, promote cancer cells to invade and metastasize. That is, the increase in tumor metastasis caused by sunitinib may be difficult to avoid because sunitinib “correctly” plays its role. The antiangiogenic resistance has been discussed recently [43–46]. Our finding suggests that there are potential risks in therapies directly targeting ECs because EC-constituted blood vessels not only provide nutrients and oxygen to the TCs but also are the most important component of the tumor microenvironment that orchestrates cancer cell behavior.

Our data may be of help in making sunitinib useful in the context of metastatic breast cancer. As shown in this study, sunitinib can stimulate the endothelial cells secreting inflammatory chemokines, which will attract the tumor cells for they often express chemokine receptors [27]. Therefore, inhibiting the chemokine signaling cascade, such as inhibiting SASP using a JAK inhibitor in combination with sunitinib, may block the tumor-endothelial cell interactions and immune cell recruitment, resulting in inhibition of metastasis. Blocking the monocyte recruitment using a CCR2 inhibitor or neutralizing antibody may be another effective way to improve the efficacy of sunitinib. As reported by Pollard’s group, blocking CCL2/CCR2 interaction effectively inhibited the recruitment of metastasis-associated macrophages and inhibited breast cancer metastasis [47, 48].

## Conclusions

Our data showed that sunitinib induced EC senescence that promotes cancer cells to invade and metastasize. According to these results, although sunitinib was designed to target the EC directly, the increase in tumor metastasis may ironically be due to sunitinib “correctly” playing its role. Thus, we should carefully weigh the pros and cons before using sunitinib and other antiangiogenic drugs that directly target the ECs.

## Data Availability

Yes.
